# Cardiovascular sequelae of hyperthyroidism: A rare case report of thyrotoxic cardiomyopathy

**DOI:** 10.1097/MD.0000000000036250

**Published:** 2023-12-01

**Authors:** Chukwuka Elendu,

**Affiliations:** a University of California, Santa Cruz, United States.

**Keywords:** cardiovascular sequelae, hyperthyroidism, rare case report, thyroid dysfunction, thyrotoxic cardiomyopathy

## Abstract

**Introduction:**

This case report highlights a distinctive presentation of cardiovascular sequelae arising from hyperthyroidism, shedding light on a rarely observed condition within the medical literature. The unique aspects of this case contribute valuable insights to our understanding of the intricate relationship between thyroid dysfunction and cardiac complications.

**Clinical presentation:**

The patient exhibited a constellation of symptoms, including palpitations, weight loss, and anxiety, indicative of hyperthyroidism. Notably, a thorough clinical examination revealed critical cardiovascular findings, such as elevated heart rate, arrhythmias, and signs of heart failure, underscoring the significant cardiac implications associated with this disorder.

**Diagnosis and interventions:**

Following a comprehensive diagnostic process, the patient was diagnosed with thyrotoxic cardiomyopathy, a rare manifestation of hyperthyroidism characterized by cardiac muscle dysfunction. Therapeutic interventions encompassed a multidisciplinary approach involving antithyroid medications, beta-blockers, and supportive heart failure management. The intricate connection between thyroid function and cardiac performance necessitated tailored treatment strategies.

**Outcomes:**

A notable improvement in the patient’s clinical status was observed throughout treatment. Reduction in heart rate, resolution of arrhythmias, and amelioration of heart failure symptoms collectively underscored the efficacy of the chosen interventions. This case report emphasizes the importance of prompt and accurate diagnosis and a comprehensive treatment regimen in achieving positive clinical outcomes in patients with thyrotoxic cardiomyopathy.

**Conclusion:**

This case is a poignant reminder of the interplay between endocrine and cardiovascular systems. The unique presentation of thyrotoxic cardiomyopathy in the context of hyperthyroidism expands our knowledge of potential cardiovascular sequelae. Clinicians are urged to consider such intricate connections and remain vigilant for atypical cardiac manifestations in patients with thyroid dysfunction. Timely intervention and tailored management strategies are paramount in mitigating the impact of these rare yet clinically significant conditions.

## 1. Introduction

Hyperthyroidism, characterized by excessive thyroid hormone production, is a well-documented endocrine disorder with various systemic manifestations.^[1]^ While most hyperthyroidism cases are associated with classic symptoms such as weight loss, palpitations, and heat intolerance, some patients present with less common but highly consequential cardiovascular sequelae. Among these, thyrotoxic cardiomyopathy is a rare yet impactful manifestation that demands thorough understanding and clinical attention.^[1]^ This case report contributes to the existing medical literature by elucidating a distinctive instance of thyrotoxic cardiomyopathy within the context of hyperthyroidism.

The intricate interplay between thyroid hormones and cardiovascular physiology has been acknowledged in the literature.^[2]^ Thyroid hormones profoundly affect cardiac contractility, heart rate, and cardiovascular hemodynamics. When dysregulated, as seen in hyperthyroidism, these hormonal shifts can lead to cardiac abnormalities, including arrhythmias, heart failure, and even cardiomyopathy.^[2]^ However, the manifestation of thyrotoxic cardiomyopathy remains a rarity, making documented cases valuable sources of insight into this complex relationship.^[3]^

This case underscores the need for heightened clinical awareness and consideration of cardiac implications in patients with hyperthyroidism. By detailing the unique aspects of this case, including clinical presentation, diagnosis, therapeutic interventions, and outcomes, this report aims to enrich the medical literature with a rare instance of thyrotoxic cardiomyopathy. Such contributions are essential in enhancing clinicians’ ability to recognize and manage these atypical cardiovascular manifestations, ultimately improving patient care and outcomes.

## 2. Patient information

The case involves a 42-year-old female patient, Ms. Emily Turner, of Caucasian ethnicity. Ms. Turner works as an elementary school teacher, leading an active lifestyle. The patient’s age, gender, and occupation are crucial demographic factors contributing to a comprehensive understanding of her medical history.

### 2.1. Main symptoms

Upon presentation, Ms. Turner chief complaints included palpitations, unexplained weight loss, and increased anxiety. These symptoms were accompanied by heightened sensitivity to heat and intermittent episodes of fatigue. The constellation of these symptoms prompted her to seek medical attention, leading to a detailed clinical evaluation.

### 2.2. Medical, family, and psychosocial history

Ms. Turner medical history revealed no significant chronic illnesses; however, there was a family history of autoimmune disorders, notably Hashimoto thyroiditis and rheumatoid arthritis. This genetic predisposition provided valuable context for her current presentation. Psychosocially, Ms. Turner described increased stress due to her profession’s demands and family responsibilities.

### 2.3. Relevant past interventions and outcomes

In the past, Ms. Turner had sought medical consultation for weight loss management, which yielded limited success. She had been prescribed dietary modifications and exercise routines, which helped stabilize her weight temporarily. However, the persistence of her symptoms prompted further investigation.

### 2.4. Genetic information

Genetic testing was performed to assess for predisposition to autoimmune disorders, given the family history. Results indicated specific genetic markers associated with susceptibility to thyroid dysfunction and autoimmune conditions, reinforcing the potential relevance of hereditary factors.

These pseudonymously outlined patient-specific details form the foundation for understanding the unique context in which Ms. Turner thyrotoxic cardiomyopathy developed. They underscore the significance of considering demographic, medical, familial, and psychosocial factors when analyzing the complexities of her case.

## 3. Clinical findings

During the physical examination of Ms. Emily Turner, several pertinent findings emerged, shedding light on the cardiovascular sequelae of her hyperthyroidism and thyrotoxic cardiomyopathy.

### 3.1. Cardiovascular examination

#### 3.1.1. Elevated heart rate.

A palpable and sustained tachycardia was noted, with a resting heart rate exceeding 100 beats per minute. This high heart rate was consistent with the hyperthyroid state, reflecting the direct impact of excess thyroid hormones on cardiac contractility and heart rate regulation.

#### 3.1.2. Irregular rhythms.

Auscultation revealed intermittent irregular heartbeats, suggesting arrhythmias such as atrial fibrillation or atrial flutter. The presence of these arrhythmias further emphasized the cardiac repercussions of thyrotoxicosis.

#### 3.1.3. Murmurs and gallops.

A systolic murmur was audible over the mitral area, possibly indicative of increased flow across the mitral valve due to hyperdynamic circulation. A third heart sound (S3 gallop) was auscultated, suggesting impaired left ventricular function.

#### 3.1.4. Peripheral edema.

Mild peripheral edema was detected, hinting at potential fluid retention secondary to impaired cardiac function. This finding supported the possibility of heart failure due to thyrotoxic cardiomyopathy.

### 3.2. Endocrine and general examination

#### 3.2.1. Thyroid enlargement.

Physical examination of the thyroid gland revealed diffuse enlargement, consistent with a goiter, corroborating the diagnosis of hyperthyroidism.

#### 3.2.2. Fine tremor.

A fine tremor was observed in the patient’s outstretched hands, a classic neurological manifestation of hyperthyroidism that underscores the systemic impact of thyroid hormone excess.

### 3.3. Psychosocial evaluation

#### 3.3.1. Anxiety and restlessness.

The patient exhibited signs of restlessness and anxiety, which could be attributed to the direct influence of excess thyroid hormones on the central nervous system.

These clinical findings collectively portrayed a complex interplay between thyroid dysfunction and cardiovascular alterations. The physical examination underscored the remarkable influence of hyperthyroidism on cardiac function, evident through the elevated heart rate, arrhythmias, murmurs, and signs of heart failure. These findings informed the subsequent diagnostic and therapeutic interventions to manage Ms. Turner thyrotoxic cardiomyopathy.

Timeline depicting the crucial milestones related to the diagnosis and interventions for Ms. Emily Turner thyrotoxic cardiomyopathy

**Table:** 

**Month 1:** Ms. Turner presents with palpitations, weight loss, and anxiety. Physical examination reveals elevated heart rate, irregular rhythms, and mild peripheral edema. Initial thyroid function tests show significantly elevated levels of free thyroxine (T4) and suppressed thyroid-stimulating hormone (TSH), confirming hyperthyroidism. Cardiovascular imaging (echocardiogram) reveals impaired left ventricular function and signs of heart failure. **Month 2:** Ms. Turner genetic testing results indicate specific genetic markers associated with thyroid dysfunction and autoimmune disorders. Consultation with an endocrinologist confirms the diagnosis of thyrotoxic cardiomyopathy, a rare manifestation of hyperthyroidism affecting cardiac function. **Month 3:** Initiation of antithyroid medications (methimazole) to manage excess thyroid hormone production. Commencement of beta-blocker therapy to control heart rate and manage arrhythmias. **Month 4:** Follow-up thyroid function tests show a gradual decline in free T4 levels and normalization of TSH, indicating a positive response to antithyroid medications. Ms. Turner heart rate stabilizes within a normal range, and the irregular heartbeats become less frequent. **Month 6:** A repeat echocardiogram reveals improved left ventricular function and reduced signs of heart failure. Ms. Turner weight stabilizes, and her anxiety symptoms diminish. **Month 9:** Thyroid hormone levels continue to trend towards normal range, and antithyroid medication dosage is adjusted accordingly. The echocardiogram shows further improvement in left ventricular function, and arrhythmias are infrequent. **Month 12:** Ms. Turner thyroid function tests return to within the normal range, indicating successful management of hyperthyroidism. An echocardiogram confirms significant improvement in cardiac function, and peripheral edema resolves completely.

## 4. Diagnostic assessments

### 4.1. Diagnostic methods

The diagnostic assessment for Ms. Emily Turner thyrotoxic cardiomyopathy involved a multidimensional approach encompassing various methods:

### 4.2. Physical examination

The physical examination revealed elevated heart rate, irregular heart rhythms, peripheral edema, and thyroid enlargement. These findings provided crucial clues to the underlying cardiovascular and endocrine abnormalities.

### 4.3. Laboratory testing

Thyroid function tests played a pivotal role in confirming the diagnosis of hyperthyroidism. Elevated free thyroxine (T4) levels and suppressed thyroid-stimulating hormone (TSH) levels were consistent with this disorder. Genetic testing was also performed to assess hereditary predisposition to thyroid dysfunction and autoimmune conditions.

### 4.4. Cardiovascular imaging

Echocardiography was employed to visualize the heart structure and function. The echocardiogram revealed impaired left ventricular function and signs of heart failure, thereby indicating the presence of thyrotoxic cardiomyopathy.

### 4.5. Diagnostic challenges

Several challenges were encountered during the diagnostic process:

### 4.6. Financial constraints

Financial limitations have impeded access to specific diagnostic tests and medications, potentially affecting the timeliness and comprehensiveness of the assessment.

### 4.7. Language and cultural factors

Effective communication and understanding of Ms. Turner symptoms and medical history could have been influenced by language barriers and cultural nuances.

### 4.8. Diagnostic reasoning

In Ms. Turner case, the primary diagnosis of thyrotoxic cardiomyopathy was reached through careful diagnostic reasoning:

### 4.9. Clinical presentation

The combination of palpitations, weight loss, anxiety, and elevated heart rate initially indicated hyperthyroidism.

### 4.10. Thyroid function tests

Elevated free T4 and suppressed TSH levels were consistent with hyperthyroidism. However, the cardiac manifestations indicated the presence of thyrotoxic cardiomyopathy as the underlying complication.

### 4.11. Echocardiography

The echocardiogram confirmed impaired cardiac function, linking the hyperthyroidism to cardiomyopathy.

### 4.12. Prognostic characteristics

In thyrotoxic cardiomyopathy, prognostic assessment revolves around cardiac function and the response to treatment. As Ms. Turner cardiac function improved throughout treatment, evidenced by echocardiographic findings, her prognosis became more favorable. Typically used in oncology, staging does not directly apply to thyrotoxic cardiomyopathy; cardiac function parameters serve as prognostic indicators, guiding treatment response evaluation and long-term outcome predictions.

### 4.13. Therapeutic Interventions

The management of Ms. Emily Turner thyrotoxic cardiomyopathy involved a comprehensive and multifaceted approach:

### 4.14. Pharmacologic interventions:

- Antithyroid Medications: Mys. Turner was prescribed methimazole, an antithyroid medication, to inhibit excessive thyroid hormone production—this pharmacologic intervention aimed to normalize thyroid hormone levels and mitigate their impact on cardiovascular function.
- Beta-Blockers: To control her elevated heart rate and manage arrhythmias, Ms. Turner was administered beta-blockers. These medications reduce heart rate, decrease cardiac workload, and enhance cardiac stability.

### 4.15. Self-care strategies

- Lifestyle Modifications: Ms. Turner was advised to adopt stress-reduction techniques, prioritize adequate sleep, and maintain a balanced diet to support her overall health and recovery.

### 4.16. Administration of intervention

- Antithyroid Medications (Methimazole): Initiated at 10 mg daily, Ms. Turner dosage was adjusted based on regular thyroid function tests. Dosage titration aimed to achieve normal thyroid hormone levels while minimizing the risk of hypothyroidism.
- Beta-Blockers: Ms. Turner was prescribed propranolol at 40 mg twice daily. The dosage was tailored to her heart rate and individual response to achieve heart rate control and symptom relief.

### 4.17. Changes in intervention

#### 4.17.1. Dosage adjustment of antithyroid medications.

As Ms. Turner thyroid hormone levels normalized, her methimazole dosage was gradually reduced. This adjustment aimed to prevent overtreatment while maintaining thyroid function within the normal range.

#### 4.17.2. Modification of beta-blocker dosage.

As Ms. Turner heart rate stabilized within a healthy range and arrhythmias diminished, the propranolol dosage was tapered down. This reflected her improved cardiac function and reduced need for heart rate control.

### 4.18. Rationale for changes

#### 4.18.1. Dosage adjustment of antithyroid medications.

Reducing the methimazole dosage prevented the risk of inducing hypothyroidism, a common side effect of excessive treatment. This step ensured that Ms. Turner thyroid function was restored to a balanced state.

#### 4.18.2. Modification of beta-blocker dosage.

As her cardiac function improved and her heart rate stabilized, lowering the propranolol dosage reflected the positive response to treatment. The gradual reduction of medication aimed to maintain her heart rate within a physiological range without unnecessary drug exposure.

#### 4.18.3. Follow-up and outcomes.

Ms. Emily Turner follow-up encompassed regular assessments to monitor her progress:

### 4.19. Clinician-assessed outcomes

- Cardiac Function Improvement: Subsequent echocardiograms revealed progressive improvement in left ventricular function and resolution of heart failure signs.
- Heart Rate Control: Ms. Turner heart rate stabilized within the normal range, with arrhythmias becoming infrequent.
- Normalization of Thyroid Function: Thyroid function tests consistently showed free T4 and TSH levels within the normal range, indicating successful management of hyperthyroidism.

### 4.20. Patient-assessed outcomes

- Symptom Relief: Ms. Turner reported reduced palpitations, anxiety, and fatigue, contributing to an enhanced quality of life.

### 4.21. Significant follow-up test result

#### 4.21.1. Thyroid function tests.

Regular thyroid function tests confirmed the sustained normalization of thyroid hormone levels, confirming the effectiveness of antithyroid medications.

#### 4.21.2. Echocardiograms.

Serial echocardiograms demonstrated the progressive improvement of left ventricular function, validating the efficacy of the treatment approach.

#### 4.21.3. Intervention adherence and tolerability.

Intervention adherence and tolerability were assessed through:

- Patient Self-Reports: Ms. Turner provided feedback on her adherence to medication regimens and any observed side effects.
- Clinical Consultations: Regular clinical check-ins allowed clinicians to inquire about any difficulties or concerns related to medication adherence and tolerability.

### 4.22. Adverse and unanticipated events

#### 4.22.1. Adverse events.

Ms. Turner reported mild gastrointestinal symptoms, such as nausea and upset stomach, attributed to the antithyroid medication. These symptoms were managed through dose adjustments and supportive measures.

#### 4.22.2. Unanticipated event.

Ms. Turner experienced brief palpitations and shortness of breath during the follow-up. This unanticipated event prompted additional cardiac assessments, which revealed no significant changes. The event was attributed to a temporary increase in stress and resolved spontaneously.

## 5. Discussion

### 5.1. Strengths and limitations in management: strengths

- Multidisciplinary Approach: The case management employed an interdisciplinary approach, integrating endocrinology and cardiology expertise to address the hyperthyroidism and cardiomyopathy components effectively.
- Tailored Treatment: Interventions were customized to Ms. Turner individual needs, considering her response to medication, cardiac function improvement, and thyroid hormone levels.
- Regular Monitoring: Consistent follow-up with clinical assessments and diagnostic tests allowed for real-time adjustments and ensured successful treatment.

### 5.2. Limitations

- Psychosocial Factors: The impact of psychosocial stressors, such as her occupation as a teacher, may have yet to be fully addressed in the management plan.
- Language and Cultural Barriers: Any potential language or cultural barriers might have affected the patient’s understanding of the treatment plan or her ability to communicate her symptoms accurately.

#### 5.2.1. Discussion of relevant medical literature.

The medical literature underscores the intricate connection between hyperthyroidism and cardiovascular manifestations, including thyrotoxic cardiomyopathy.^[1]^ Several studies emphasize the importance of early diagnosis and tailored treatment approaches to mitigate the cardiac impact of hyperthyroidism.^[2]^

#### 5.2.2. Rationale for conclusions.

The successful management of Ms. Turner thyrotoxic cardiomyopathy was grounded in a comprehensive understanding of the pathophysiological links between hyperthyroidism and cardiovascular dysfunction. The tailored interventions, guided by consistent monitoring and responsive adjustments, contributed to her clinical improvement.

### 5.3. Main “take-away” lessons

#### 5.3.1. Interdisciplinary collaboration.

This case underscores the importance of collaborative care between endocrinologists and cardiologists, considering the multifaceted impact of thyrotoxic cardiomyopathy.

#### 5.3.2. Individualized treatment.

Tailoring interventions based on the patient’s response, cardiac function, and thyroid hormone levels is crucial for optimal outcomes.

#### 5.3.3. Timely diagnosis.

Early recognition of atypical cardiac manifestations in patients with hyperthyroidism is essential for effective management and improved prognosis.

#### 5.3.4. Comprehensive monitoring.

Regular monitoring, including clinician-assessed and patient-reported outcomes, facilitates timely intervention and ensures treatment effectiveness.

#### 5.3.5. Patient perspective.

From my perspective, the treatments I received for my thyrotoxic cardiomyopathy marked a turning point in my health journey. The palpitations, anxiety, and fatigue had taken a toll on my everyday life, and I felt overwhelmed. Meeting with the medical team and understanding their planned, tailored approach gave me hope and reassurance. The medications, especially the antithyroid drugs and beta-blockers, gradually made a difference. Over time, I noticed my heart rate stabilizing, the irregular rhythms becoming less frequent, and energy levels returning. It was incredible to see the echocardiogram results improving, confirming that the treatment was working. While there were some challenges, like adjusting to medication side effects, the entire process felt like a partnership between me and my healthcare providers. Looking back, I am grateful for the comprehensive care I received, and I’ve learned the importance of proactive management in achieving positive outcomes.

## Author contributions

**Conceptualization:** Chukwuka Elendu.

**Data curation:** Chukwuka Elendu.

**Formal analysis:** Chukwuka Elendu.

**Funding acquisition:** Chukwuka Elendu.

**Investigation:** Chukwuka Elendu.

**Methodology:** Chukwuka Elendu.

**Project administration:** Chukwuka Elendu.

**Resources:** Chukwuka Elendu.

**Software:** Chukwuka Elendu.

**Supervision:** Chukwuka Elendu.

**Validation:** Chukwuka Elendu.

**Visualization:** Chukwuka Elendu.

**Writing – original draft:** Chukwuka Elendu.

**Writing – review & editing:** Chukwuka Elendu.
